# Supplementary material for: Complex‐centric proteome profiling by SEC‐SWATH‐MS
Source: Mol Syst Biol. 2019 Jan 14;15(1):e8438. doi: 10.15252/msb.20188438 (PMC6346213; doi:10.15252/msb.20188438)
Supplement: Supplementary file 7 — Dataset EV6 [file MSB-15-e8438-s007.zip › feature_plots_bioplex/Q13277.pdf]

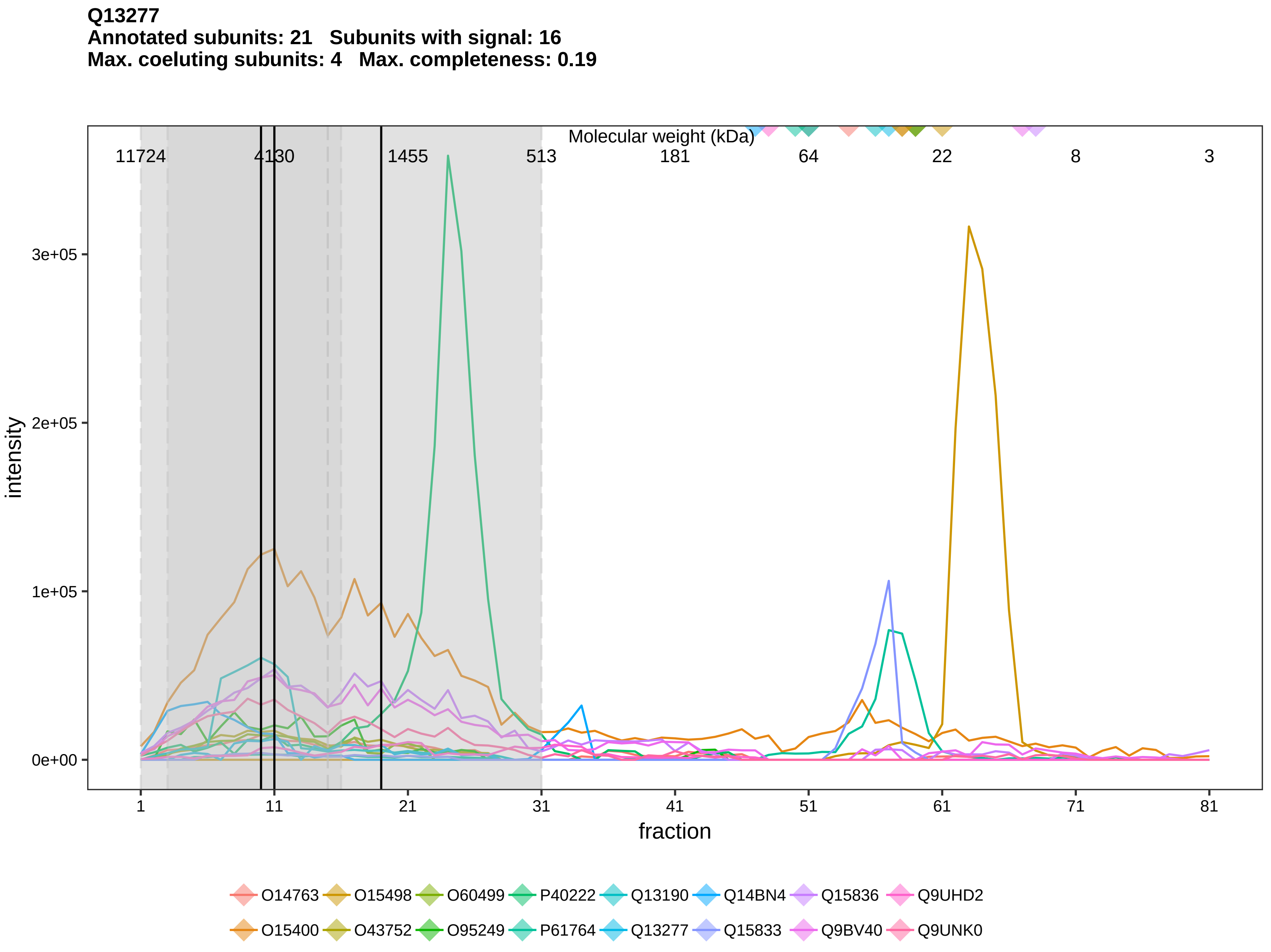

Q13277
Annotated subunits: 21 Subunits with signal: 16
Max. coeluting subunits: 4 Max. completeness: 0.19
Molecular weight (kDa)
11724
4130
1455
513
181
64
22
8
3
intensity
3e+05
2e+05
1e+05
0e+00
1
11
21
31
41
51
61
71
81
fraction
O14763
O15498
O60499
P40222
Q13190
Q14BN4
Q15836
Q9UHD2
O15400
O43752
O95249
P61764
Q13277
Q15833
Q9BV40
Q9UNK0
